# A controlled trial of the effectiveness of a diabetes education programme in a multi-ethnic community in Glasgow [ISRCT28317455]

**DOI:** 10.1186/1471-2458-6-134

**Published:** 2006-05-18

**Authors:** Hamid R Baradaran, Robin P Knill-Jones, Sunita Wallia, Alison Rodgers

**Affiliations:** 1 Medical Education & Development Centre, Iran University of Medical Sciences, Tehran, 14496, Iran; 2 Public Health and Health Policy Section, University of Glasgow, Glasgow G12 8RZ, UK; 3 120–130 William Street Clinic Glasgow G3 8UR, UK; 4 Rutherglen Primary Care Centre, 130 Stonelaw Road, NHS Greater Glasgow, UK

## Abstract

**Background:**

Epidemiologic data have shown that the prevalence of Type 2 diabetes varies with ethnic origin. Type 2 diabetes is up to four times more common in British South Asians than in the indigenous white population. The aim of this study was to develop a culturally appropriate educational intervention programme for South Asians with Type 2 diabetes. We then investigated whether this intervention could produce an improvement, and finally whether any improvement was greater than background changes in knowledge in comparison groups.

**Methods:**

A multi-site prospective, randomised controlled study was conducted in all day care centres and three general practice registers with high proportion patients from different ethnic minority groups in Glasgow, Scotland. The intervention consisted of 18 educational sessions in 6 separate programmes. A modified questionnaire was used to measure the knowledge, attitudes, and practice of diabetes before and after intervention.

**Results:**

Baseline assessment showed that Indian and Pakistani subjects had less knowledge about diabetes, regarded the disease less seriously, and had a lesser understanding of the relationship between control and complications than the white population. No differences in initial responses were found between those who completed the second assessment and those who did not. The intervention group showed significant improvements in scores for Knowledge (+12.5%); Attitudes toward seriousness (+13.5%), complications (+8.1%), Practice (+20.0%). However there were also changes in the ethnic control group scores; respectively +5.0%, +16.3% (significant P < 0.001), +1.5%, +1.7%. The single white control group also showed some improvements; respectively +12.2%, +12.4% (P = 0.04), +6.0%, +25.0% (P = 0.007), but the differences in improvement between these two control groups were not significant. Overall, the improvement seen was similar in both intervention and ethnic control groups and there was no significant difference in the amount of change (P = 0.36 CI -0.9 to +2.6).

**Conclusion:**

This study has shown that conducting a culturally-competent educational intervention in patients with Type 2 diabetes from ethnic minority groups is feasible and can improve their knowledge and attitudes and practice. However there was no net benefit compared with the control group.

## Background

The prevalence of Type 2 diabetes is predicted to rise over the next few decades which will pose a major public health challenge [1]. Epidemiologic data have shown that the prevalence of Type 2 diabetes varies with ethnic origin. People originating from the Indian subcontinent ('South Asians') who have settled in other parts of the world seem to be particularly susceptible to Type 2 diabetes [2-6]. Type 2 diabetes is up to four times more common in British South Asians than in the indigenous white population; in the UK one in five older South Asians have diabetes [7]. It is recognized that good education is a keystone in diabetes management, and it is worrying that some studies of different South Asian communities in Britain have shown that they generally know less about diabetes and its management than comparable white subjects [8-10]. Our initial aim was to assess baseline levels of knowledge, attitudes, and some aspects of self-management of diabetes care in a representative sample of South Asian people with Type 2 diabetes in Glasgow, UK. Then we used the baseline results to help develop a culturally appropriate (competent) educational intervention programme for these groups. We then investigated whether this intervention could produce an improvement, and finally whether any improvement was greater than background changes in knowledge in comparison groups.

## Methods

### Study setting and population

South Asian people (defined as anyone of Indian subcontinent origin, whether from Pakistan, India, Bangladesh or Sri Lanka) with Type 2 diabetes over the age of 30 years were identified from day care centres catering for significant numbers of ethnic minorities, and three general practices which had more than 70% of their patients from different ethnic minority groups. Letters were sent to all individuals via their general practitioner and/or day care staff inviting them to participate in this study. If there was no response, patients were telephoned by the relevant care staff. One hundred and forty five patients, [Pakistani (n = 85), Indian (n = 33), White (n = 27)] were available and willing to participate. Before collecting the baseline data written informed consent was obtained from all patients. For those who were unable to read any language the consent form and details of intervention were read and explained by bilingual staff, and then their agreement was obtained. The study was approved by the Greater Glasgow Community/Primary Care local Research Ethics Committee.

### Study design

**Figure 1:**
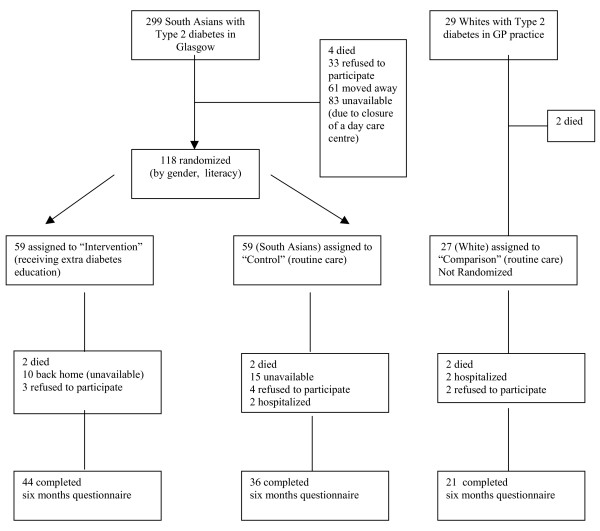
Outline the flow of patients from baseline to 6-month follow up.

**Figure 2:**
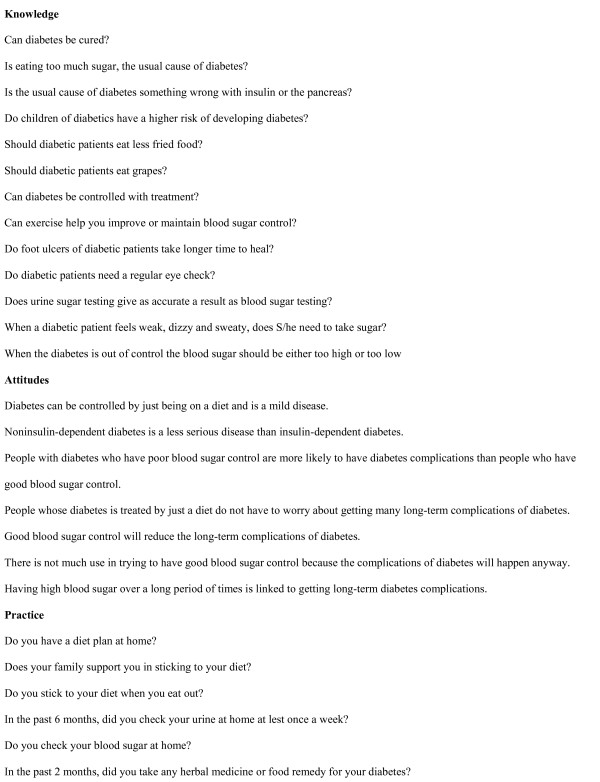
Questions asked to assess knowledge, attitudes and practice about diabetes.

We conducted a controlled trial in South Asians, and also studied a white group for comparative purposes. The South Asian group was divided based on gender, then each stratum was further divided based on their reading ability in any language. Finally minimization was performed for the resulting four strata to allocate individuals into intervention (n = 59) and control groups (n = 59). The White comparison group (n = 27) was separate (Figure 1). Before randomisation a questionnaire at the start of the study (baseline) assessed the patients' knowledge, attitudes, and practice, about diabetes. The development, content and structure of the questionnaire have been fully described elsewhere [10]. The outline of the questions used is shown in figure 2. After randomisation, baseline results were used to develop an appropriate, culturally competent, educational intervention about diabetes which was carried out by a bilingual health educator team (one podiatrist and one dietician). The baseline questionnaire was then re-administered at the end of the study to all subjects (control and intervention) with change in score as the primary outcome, and difference in changes in score as the secondary outcome.

### Intervention

Despite the existence of several types of educational programmes for Type 2 diabetes, there was no specific standard for diabetes education, particularly for ethnic minority groups in developed countries available during this study. Therefore the questionnaire responses were analyzed and used to help develop the educational tool for sessions. In addition, recommendations of national and international diabetes organisations, such as Diabetes UK, and also information from previous studies were utilised for this purpose [11]. The educational intervention was carried out in day care centres and in GP's surgeries. It consisted of three sessions, one dietician-led sessions of about one hour's duration, and one podiatrist-led session of about one and a half hours' duration. They were carried out and completed within three months. The format of the educational programme was based on group education; the size of each group being between 6 and 12. The learning process in the groups was a combination of didactic elements (lecture) and interactive group discussion. During classes patients were asked to discuss some of their experiences and problems. Then the educator explained issues relevant to diabetes such as pathophysiology and cause of diabetes, short and long-term complications of diabetes, blood glucose control, recommendations for appropriate lifestyle changes (e.g., exercise, smoking cessation), nutrition recommendations (culturally appropriate), foot care, instruction about when and how to contact the physician or other members of the health care team when the patients were unable to solve acute problems themselves. The educators also used some simple support material including visual aids, food examples (real, models or packages, as applicable). Additionally some booklets and leaflets about diabetes, diet and foot care, translated into Urdu, Punjabi and Hindi, provided by Diabetes UK, were given to each patient after each session. Furthermore the educational team used an information video (25 minutes) in the second session which was recorded in their language.

Both team members were able to speak Punjabi, the preferred language that most patients speak at home. The Punjabi language is a common language even for those who also speak Hindi or Urdu. A total of 18 educational sessions in 6 separate programmes were carried out. Since patients had different religions and came from different cultures three programmes were implemented only for women, two programmes for men, and one programme for mixed women and men. In day care centres suitable rooms properly furnished and equipped with audio-visual instruments, a white board, and posters were used for this programme. In the GP's surgery one room was allocated for the diabetes educational intervention. This room was comfortable for patients and had similar equipment.

### Analysis

To analyse data both descriptive and analytic statistics were applied. For descriptive analysis results are expressed as numbers, percentages, mean (± SD and 95% CI). Differences between the characteristics of the patient groups were compared, using *Chi-Squared *(for categorical variables) and *t-test *(for continuous variables). *One-way analysis of variance *(ANOVA) was applied to examine differences in variables between the three groups at baseline. *Paired t-test *was used to examine outcome by comparing baseline and follow-up assessments. The two-sample t-test was used to examine the changes in scores when comparing the intervention group with the ethnic control group and the changes in the two control groups. SPSS, version 11.5 for Windows (SPSS, Chicago, IL) was used.

## Results

Of 299 identified patients in ethnic minorities with diabetes in the day care centres and the three GP practices, 61 had the letters returned, and an additional 83 became unavailable as one day care centre closed, meaning that access for the intervention became impossible. There were 33 who refused to participate, and four died during the preparation of the intervention. 118 were available for randomisation. In addition there were 29 white patients with Type 2 diabetes in two of the GP practices who were recruited as a comparison group. Of these two died before the initial assessment. The characteristics of the 118 randomised patients and the 27 patients in the white comparison group are shown in Table 1. There were no statistically significant differences at baseline between the intervention and control groups except for marital status (fewer white group were married, (P = 0.03), and self-reported reading ability, illiteracy being confined to the ethnic groups (P = 0.01).

Of the 145 (including the white group), only one hundred and one could be re-interviewed at 6 months. At the time of follow-up, out of 145 patients six (4.1%) were dead, four were hospitalised, twelve refused to participate in the second questionnaire, and 25 (17.2%) patients were unavailable. Some of these patients had returned to their home country but the proportion was unknown. Details are given in figure 1. To assess the possibility of a selective bias introduced by loss of patients the dropped out group and the group who completed the study were compared for their baseline findings. No differences were found.

All mean scores were significantly higher after the intervention (Table 2). For knowledge the score in the intervention group increased after the educational intervention by 12.5% from the baseline knowledge score, (P = 0.04). This group also showed more positive attitudes towards seriousness and value of tight control of diabetes after the educational intervention (increases of 13.5%, P = 0.005 and 8.1%, P = 0.05 respectively). Finally the educational intervention had a considerable effect on practice about diabetes in this group (20% increase from the baseline score, (P = 0.005).

In the ethnic control group there was no statistically significant difference in three of the four scores between baseline and follow-up (Table 3). However there was a significant improvement of 16.3% in attitudes towards seriousness of diabetes (P = 0.001).

In the white comparison group who did not have an intervention there was a significant increase in two scores (Table 4),-attitudes towards seriousness (12%), and practice of Type 2 diabetes (10%).

The amount of change in the four scores was compared for the ethnic intervention and control group in Table 5. The improvement seen was similar in both groups and there was no significant difference in the amount of change. However in three scores the change was greater in the intervention group than the control group. Table 6 compares the amount of change in the two comparison groups. It tends to be greater changes in the white comparison group compared to the ethnic control group, but the differences were not significant.

## Discussion

Overall, the results of educational interventions aimed at patients with Type 2 diabetes are difficult to interpret. The diabetes education intervention for ethnic minority groups in Glasgow demonstrated that South Asians with Type 2 diabetes could improve their knowledge and change their attitudes about Type 2 diabetes. The results showed an increase in diabetes knowledge and changes of attitudes, particularly regarding seriousness of Type 2 diabetes in the intervention group. However there was also an improvement in the ethnic control group during the period of the study, and also in the white comparison group. This finding is not unusual, as other studies have shown similar effects in control groups [12-14].

There are several reasons why the knowledge, attitudes, and practice of diabetes in the control group have improved. We looked at potential biases. Those who dropped out from the ethnic group might have been different from those who stayed within the study. However, our analysis showed no difference in starting scores in those members of the ethnic control group who stayed in the study compared to those who dropped out. Some increased knowledge during the study in the control group subjects, and the white comparison group, might have resulted from merely completing the study questionnaires. Subjects' questions were answered frankly during the interview, regardless of whether subjects were in the intervention or the control group. Completing the questionnaire may have had an effect on its own. It was not considered ethical to withhold information from the control group subjects during data collection and all questions were answered. In addition, the control groups had frequent subsequent interactions with their health care providers (such as their GP or practice nurse) following the initial assessment and may have been stimulated by the study to ask further questions from their carers.

One of the issues which arises in this kind of study is contamination. This might happen particularly in day care centres where people are used to meeting weekly. The day care centres provided a good opportunity for people to discuss and exchange information with each other. Therefore, after the education sessions for the intervention groups, subjects in the intervention groups may talk to their friends in the control group and may have discussed some of the issues raised by the questionnaire. We could not control this, but in the GP practices such contamination was much less likely.

Another possible explanation might be the 'reactive (Hawthorne) effect'. This refers to the effect of being studied upon those who know they are in a study. Their knowledge of the study may influence their behaviour (they may become more interested in the topic, pay more attention to it and become biased), or they may change their behaviour simply because someone (the investigator) is taking an interest in them. When people feel that they are being tested, they feel the need to create a good impression. Alternatively if the study stimulates interest not previously felt for the topic under investigation, this may distort the results.

Finally translation is a significant issue for interviewing with non-English speakers. Although the translators were asked not to interpret any question and add much from their own views, the accuracy and validity of this procedure was difficult to measure. Translation for ethnic minority groups with different languages is not just a word by word translation. Therefore, the effect of the translator on the subject's answers and subsequent knowledge might be a further confounding factor.

The major question that must be asked is whether the intervention tested in this study was effective. It was for all four scores. However there was 'background' improvement in the ethnic control group which may have had contamination in the day care settings from the intervention group. There was also improvement in the white comparison group, all from a general practice setting where confounding was less likely, indicating that there was some improvement in knowledge in the wider population with diabetes in Glasgow at least. Intervention studies of this type are always subject to influences outwith the control of the investigators. The relative effect of the intervention might not be significant, but the absolute effect was that knowledge improved in the ethnic population with diabetes, regardless of how it was produced or induced. That improvement in understanding should lead in the end to better diabetic control.

## Conclusion

This study has provided useful information about Type 2 diabetes amongst South Asians in Glasgow. Based on the findings and experience gained through the study the following recommendations are made for diabetes care and for further research. Since the UK is a multi-cultural country, culturally competent behavioural interventions should be the focus of major national initiatives. Future research should be aimed at developing culturally appropriate outcome measures, addressing translation issues for non-English speaking populations, and exploring motivating factors and strategies for diabetes self-management. Consideration needs to be made about: how to involve people who would benefit most from diabetes education interventions in community gathering places and in the home; optimal intervention intensity; ideal providers; and how to integrate diabetes education with primary care. Further research is needed to determine the effect of diabetes educational interventions on other outcomes such as complication rates, lifestyle, cost, and quality of life.

## Competing interests

The author(s) declare that they have no competing interests.

## Authors' contributions

HRB and RPKJ developed the study design. HRB performed the data collection and analysed the data and wrote the first manuscript. SW and AR carried out the educational intervention. RPKJ participated in the editing and revising the manuscript. All authors contributed to, have read and approved the final manuscript.

## Pre-publication history

The pre-publication history for this paper can be accessed here:



## Figures and Tables

**Table 1:** The characteristics of the study sample.

	**Ethnic Intervention (n = 59)**	**Ethnic Control (n = 59)**	**White control (n = 27)**	**Total (n = 145)**	**P**
		
	**No. (%)**	**No. (%)**	**No. (%)**	**No. (%)**	
**Gender**					
Male	28 (47.5)	33 (56)	13 (48)	74 (51)	0.61
Female	31 (52.5)	26 (44)	14 (52)	71 (49)	
**Age (years)**					
Mean (SD)	57.8 (12.7)	59.2 (11.3)	58 (13.8)	58.4 (12.3)	0.81
Range	36–93	31–90	33–78	31–93	
**Duration of Diabetes (years)**					
Mean (SD)	7.9 (6.6)	10.1 (8.4)	6.2 (5.7)	8.5 (7.4)	0.06
Range	1–29	1–33	1–20	1–33	
**Years of Education**					
Mean (SD)	6.6 (5.1)	6.7 (5.1)	8.5 (2.3)	7.0 (4.7)	0.17
Range	0–16	0–18	5–14	0–18	
**Reading Ability**					
None	15 (25.4)	16 (27)	------	31 (21)	0.01
Any language	44 (74.6)	43 (73)	27 (100)	114 (79)	
**Marital status**					
Married	46 (78)	52 (88)	17 (63)	115 (79)	0.03
Never married/Separated/divorced/Widowed	13 (22)	7 (12)	10 (37)	30 (21)	
**Employment status**					
Employed	12 (20)	15 (25)	8 (30)	35 (24)	0.61
Unemployed/Retired/housewife	47 (80)	44 (75)	19 (70)	110 (76)	
**Preparing Meal**					
Cook for self	18 (30.5)	21 (36)	13 (48)	52 (36)	0.28
Cook by others	41 (69.5)	38 (64)	14 (52)	93 (64)	

**Table 2:** The mean KAP scores in the Intervention group, before and after the educational intervention.

	**Pre-test**	**Post-test**	**Change**	**95% CI**	**P**
		
	Mean (SD)	Mean (SD)	Mean (SD)		
**Knowledge**					
	13.6 (4.8)	15.3 (4.7)	1.7 (5.4)	0.1 to 3.4	**0.04**
Percent change			+ 12.5 %		
					
**Attitude towards seriousness**	8.9 (1.7)	10.1 (2.1)	1.2 (2.4)	0.3 to 2.0	**0.005**
Percent change			+13.5 %		
					
**Attitude towards complications**	13.5 (2.0)	14.6 (2.7)	1.1 (3.4)	0.02 to 2.1	**0.05**
			+8.1 %		
					
**Practice**	5.5 (2.9)	6.6 (2.6)	1.0 (2.2)	0.3 to 1.7	**0.005**
Percent change			+ 20 %		

**Table 3:** The mean KAP scores in the Ethnic control group – baseline compared to follow up.

	**Pre-test**	**Post-test**	**Change**	**95% CI**	**P**
		
	Mean (SD)	Mean (SD)	Mean (SD)		
**Knowledge**	14.2 (5.5)	14.7 (4.1)	0.4 (4.5)	-1.1 to 2.0	0.53
Percent change			+ 5.0 %		
**Attitude towards seriousness**	9.0 (1.7)	10.4 (2.4)	1.3 (2.2)	0.5 to 2.1	**0.001**
Percent change			+16.3 %		
**Attitude towards complication**	13.9 (1.9)	14.4 (2.9)	0.4 (3.0)	-0. 6 to 1.5	0.40
Percent change			+1.5%		
**Practice**	5.8 (2.7)	6.0 (2.3)	0.2 (3.2)	-0.8 to 1.3	0.63
Percent change			+ 1.7 %		

**Table 4:** The mean KAP scores in the White control group – baseline compared to follow up.

	**Pre-test**	**Post-test**	**Change**	**95% CI**	**P**
			
	Mean (SD)	Mean (SD)	Mean (SD)		
**Knowledge**					
	17.2 (5.0)	19.2 (5.2)	2.2 (7.0)	-1.1 to 5.2	0.19
Percent change			+ 12.2 %		
**Attitude towards seriousness**					
	9.6 (2.1)	10.8 (2.3)	1.2 (2.5)	0.01 to2.3	**0.04**
Percent change			+12.4 %		
**Attitude towards complication**					
	15.0 (1.9)	15.9 (2.0)	0.9 (3.0)	-0.4 to 2.2	0.19
			+6.0 %		
**Practice**					
	4.2 (2.3)	5.4 (2.2)	1.1 (1.6)	0.3 to 1.8	**0.007**
Percent change			+ 25.0 %		

**Table 5:** Comparison of the changes in scores in the ethnic intervention group compared to changes in the ethnic control group.

	**Intervention group**	**Control group**	**P**
		
	Mean change (SD)	Mean change (SD)	2 sample t-test
**Knowledge**			
	1.72 (5.4)	0.47 (4.5)	0.27
**Attitude towards seriousness**			
	1.21 (2.4)	1.38 (2.2)	0.76
**Attitude towards complication s**			
	1.13 (3.4)	0.45 (3.0)	0.38
**Practice**			
	1.02 (2.2)	0.26 (3.2)	0.23

**Table 6:** Comparison of the changes in scores in the ethnic control group compared to the white comparison (control) group.

	**Ethnic Control group**	**White Comparison group**	**P**
		
	Mean change (SD	Mean change (SD)	2 sample t-test
**Knowledge**			
	0.47 (4.5)	2.2 (7.0)	0.58
**Attitude towards seriousness**			
	1.38 (2.2)	1.2 (2.5)	0.60
**Attitude towards complication**			
	0.45 (3.0)	0.9 (3.0)	0.55
**Practice**			
	0.26 (3.2)	1.1 (1.6)	0.29
